# Ustekinumab for Extraintestinal Manifestations in Patients With Inflammatory Bowel Disease: Lessons From the TENOR Real-World Study

**DOI:** 10.1016/j.gastha.2026.101115

**Published:** 2026-09-17

**Authors:** Romain Altwegg, Miceli Corinne, Paul Carle, Sosnowiez Katia, Bessette Christel, Peyrin-Biroulet Laurent

**Affiliations:** 1 Department of Gastroenterology, Hôpital Saint-Eloi, CHU de Montpellier, Montpellier, France; 2 Department of Rheumatology, Cochin Hospital, Paris, France; 3 Department of Dermatology, CHU de Toulouse, Toulouse, France; 4 J&J Innovative Medicine, France; 5 ICON plc, Puteaux, France; 6 Department of Gastroenterology, CHRU de Nancy, Vandœuvre-lès-Nancy, France

Inflammatory bowel disease (IBD), encompassing Crohn’s disease (CD) and ulcerative colitis (UC), is a chronic, immune-mediated inflammatory condition of the gastrointestinal tract.^1^ Although gastrointestinal symptoms predominate, extraintestinal manifestations (EIMs) and immune-mediated inflammatory diseases (IMIDs) occur in 20% to 40% of patients, complicating management and reflecting a more complex, morbid disease phenotype.^2^^,^^3^

Biologic therapies have transformed IBD management, but evidence regarding their efficacy in EIM/IMID remains limited. Ustekinumab (UST), a monoclonal antibody targeting interleukin 12 and 23, is approved as second-line therapy for moderate-to-severe IBD. Emerging data suggest UST may confer benefits in EIM/IMID.^4^, ^5^, ^6^, ^7^ Assessment of EIM/IMID in clinical trials remains challenging, as emphasized by the EXTRA consensus from the International Organization for the Study of Inflammatory Bowel Diseases, which proposed standardized recommendations to harmonize evaluation and mitigate limitations.^8^

TENOR (Effec**T**iveness of ust**E**kinumab in patients with extra-i**N**testinal manifestations associated with Cr**O**hn's disease in a **R**eal-world setting) was a prospective, multicenter, non-interventional French study conducted between October 2018 and November 2022 to assess UST impact on EIM/IMID in IBD through a multidisciplinary approach involving gastroenterologists and relevant specialists (dermatologists, rheumatologists, and ophthalmologists). Eligible adults had IBD, were initiating UST according to the French summary of product characteristics after prior anti-tumor necrosis factor therapy, and had at least one suspected active EIM/IMID.

Patients underwent evaluations at baseline (gastroenterologist and specialist), week 16 (gastroenterologist), and week 24 (gastroenterologist and specialist). EIM of interest included cutaneous (pyoderma gangrenosum, erythema nodosum), articular (axial and peripheral spondyloarthritis, dactylitis, enthesitis), and ocular (uveitis) manifestations. IMID comprised psoriasis, atopic dermatitis, and hidradenitis suppurativa.

The primary endpoint was the proportion of patients achieving clinical response (CR) for at least one EIM/IMID at week 24 as assessed by specialists. CR was evaluated using standardized tools. For dermatologic manifestations, CR was defined as a ≥2-point reduction in the Physician’s Global Assessment score or a score of 0 to 1; erythema nodosum was assessed by lesion resolution. For articular involvement, CR was defined as a moderate or good response on Disease Activity Score in 28 joints for peripheral spondyloarthritis (≥0.6-point reduction), Bath Ankylosing Spondylitis Disease Activity Index50 or ≥2-point reduction for axial spondyloarthritis, and a 50% decrease or score of 0 on Leeds Enthesitis Index-6 for enthesitis. Dactylitis and uveitis were evaluated by lesion resolution. Secondary endpoints included UST efficacy on IBD, assessed using the Harvey–Bradshaw Index (HBI) for CD and the Partial Mayo Score (PMS) for UC.

The primary analysis used the Extended Primary Efficacy Analysis Set, comprising all enrolled patients with specialist-confirmed EIM/IMID and assessable primary endpoint data, irrespective of visit timing. Additional methodological details are available in the Supplementary Material. Descriptive analyses were performed using SAS version 9.4 (SAS Institute Inc, Cary, NC, USA).

A total of 109 patients were enrolled (mean age, 41.8 ± 13.7 years; female-to-male ratio, 2:1), of whom 89.9% had CD and 10.1% UC; 26.6% presented with severe disease. Mean disease duration was 12.9 ± 9.9 years for CD and 8.4 ± 5.3 years for UC. At baseline, mean HBI was 7.3 ± 3.7, and mean PMS was 4.0 ± 2.3. All patients had prior exposure to at least one anti-tumor necrosis factor. Concomitant therapy was reported in 25.7%, including 14.6% corticosteroids and 11.1% immunomodulators. The most frequent historical EIM/IMID were axial spondyloarthritis (40.4%), peripheral spondyloarthritis (33.0%), and psoriasis (16.5%). The mean time since the first EIM/IMID diagnosis was 6.6 ± 8.8 years (median, 2.5 years).

At baseline, 69.7% (76/109) of patients had specialist-confirmed EIM/IMID. Among these, the most common diagnoses were axial spondyloarthritis (47.4%; mean Bath Ankylosing Spondylitis Disease Activity Index, 6.5 ± 9.4), peripheral spondyloarthritis (27.6%; mean Disease Activity Score in 28 joints, 3.3 ± 0.9), psoriasis (22.4%; 35.3% moderate to severe), and enthesitis (9.2%; mean Leeds Enthesitis Index-6, 2.0 ± 1.4). Less frequent manifestations included atopic dermatitis (5.3%), hidradenitis suppurativa (4.0%), uveitis (4.0%), erythema nodosum (2.6%), dactylitis (2.6%), and pyoderma gangrenosum (1.3%).

At week 24, CR was evaluable in 52.3% (57/109) patients. Attrition was mainly due to lack of specialist confirmation at baseline (n = 33) and absence of week 24 specialist assessment (n = 18). Consequently, statistical analyses were restricted. Approaches for handling missing data across different analyses are detailed in Figure.

**Figure:**
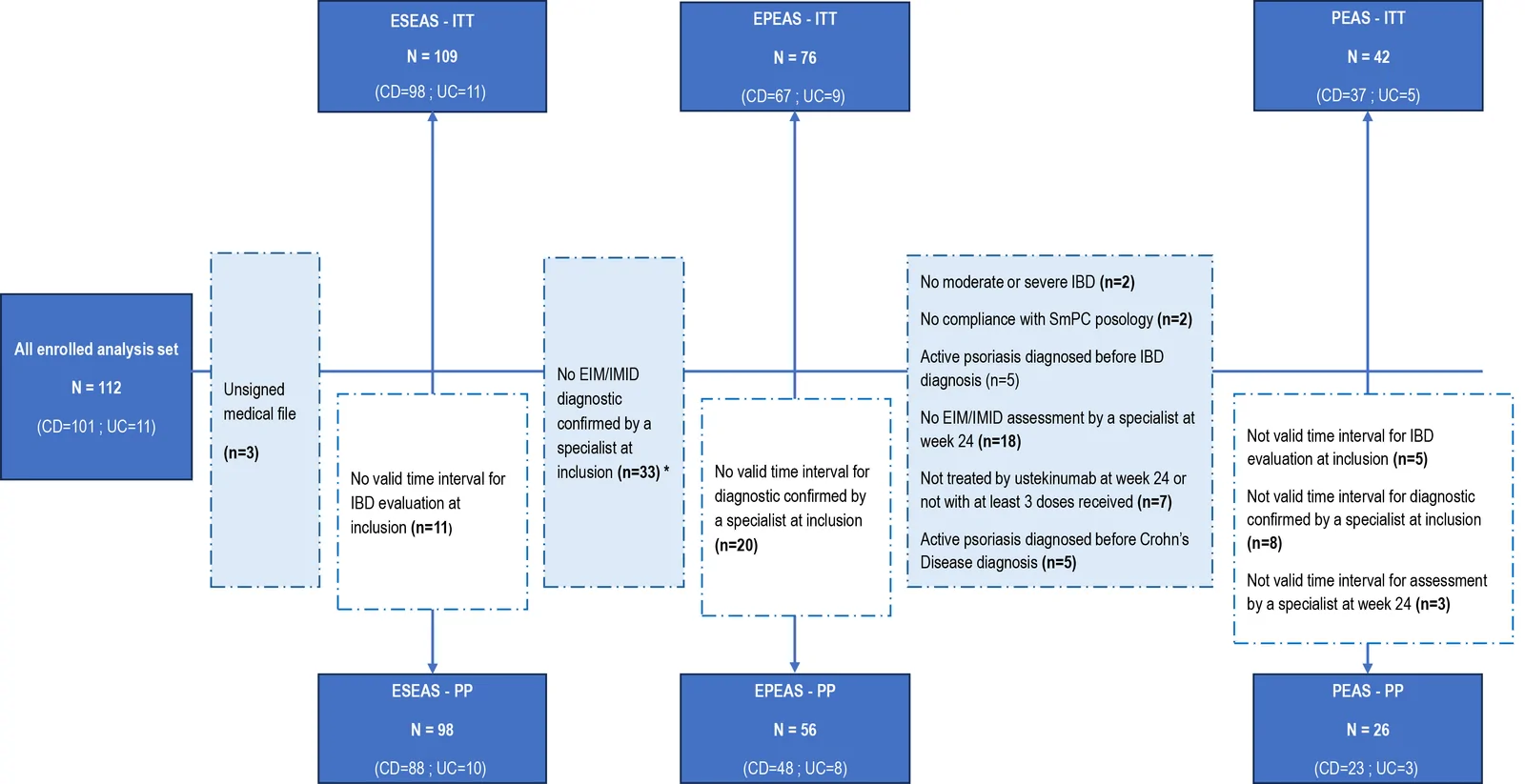
Definition of analysis sets (ITT and per protocol). EPEAS-ITT, Extended Primary Efficacy Analysis Set-intention-to-treat; EPEAS-PP, Extended Primary Efficacy Analysis Set-per protocol; ESEAS-ITT, Extended Secondary Efficacy Analysis Set-intention-to-treat; ESEAS-PP, Extended Secondary Efficacy Analysis Set-per protocol; PEAS-ITT, Primary Efficacy Analysis Set-intention-to-treat; PEAS-PP, Primary Efficacy Analysis Set-per protocol.

In Extended Primary Efficacy Analysis Set at week 24, 40.4% (23/76) achieved CR for at least one EIM/IMID. Response rates were higher for dermatologic (68.4%) than articular manifestations (21.1%). CR by manifestation is presented in Table.

**Table:** Effectiveness of Ustekinumab on the Different EIM/IMID Associated With IBD

	Confirmed diagnosis at baseline, N	Week 24 assessment			UST effectiveness N (%)
		CR^a^ N N N	No CR	Missing data	
Articular manifestations					
Axial spondyloarthritis	36	5	20	11	5/25 (20.0%)
Peripheral spondyloarthritis	22	3	13	6	3/16 (6.3%)
Enthesitis	8	2	1	5	2/3 (66.7%)
Dactylitis	2	0	1	1	0/1 (0.0%)
Cutaneous manifestations					
Psoriasis	17	9	4	4	9/13 (69.2%)
Atopic dermatitis	4	1	2	1	1/3 (33.3%)
Hidradenitis suppurativa	3	1	1	1	1/2 (50.0%)
Erythema nodosum	2	1	0	1	1/1 (100.0%)
Pyoderma gangrenosum	1	NA	NA	1	NA
Ophthalmic manifestations					
Uveitis	3	2	1	0	2/3 (66.7%)

EPEAS analysis.

DAS28, Disease Activity Score in 28 joints; EPEAS, Extended Primary Efficacy Analysis Set; LEI-6, Leeds Enthesitis Index; NA, not applicable; PGA, Physician’s Global Assessment.

a CR defined for dermatological EIM PGA improved by 2 points or nil if initial PGA 1; erythema nodosum: resolution of lesions; peripheral spondyloarthritis: reduction >0.6 on DAS28; axial spondyloarthritis: Bath Ankylosing Spondylitis Disease Activity Index50 or ≥2 reduction in score; dactylitis: resolution of lesions; enthesitis: LEI-6 at 0 or 50% reduction in score; uveitis: resolution of lesions. Several EIM/IMID per patient possible.

Mean HBI and PMS scores decreased by 3.3 ± 4.0 and 2.6 ± 2.8 points, respectively. Clinical remission was achieved in 59.8% (58/97) of CD and 80.0% (8/10) of UC patients.

Overall, 49.5% (54/109) experienced at least one adverse event (AE). Treatment-related AEs occurred in 18 patients (16.5%) and serious AEs in 12.8%, including one treatment-related serious AE (arthralgia). Seven patients (6.0%) discontinued UST due to an AE.

Although these findings suggest greater efficacy of UST in cutaneous IMID compared with articular manifestations as previously reported,^4^ definitive conclusions cannot be drawn owing to limited data interpretability.

The TENOR study highlights the complexity of evaluating EIM/IMID in IBD. A major limitation of this study is the substantial amount of missing data, resulting in limited evaluability of the endpoints. Difficulties in obtaining timely specialist consultation for non-digestive symptoms contributed to missing assessments. Consequently, CR at week 24 could only be assessed in approximately half of the enrolled patients, which restricted the interpretability of findings. Strict assessment windows also contributed to substantial missing data in the per-protocol analysis.

The limited availability of specialist evaluations primarily reflects real-world challenges in coordinating multidisciplinary care, including limited access to specialists, constrained visit schedules, and the need for multiple assessments across heterogeneous EIM/IMID. Additional contributors included lack of specialist confirmation at baseline and missed follow-up evaluations.

In some cases, specialist evaluation did not corroborate the gastroenterologist’s initial suspicion. The lack of standardized EIM/IMID definitions may limit clarity and comparability.

Study conduct was further challenged by the COVID-19 pandemic, which may have affected both patient access to care and completion of specialist assessments.

Consequently, analyses remained largely descriptive and should be interpreted with caution. Recruitment of rarer manifestations was limited, precluding meaningful interpretation for these conditions.

These difficulties mirror those reported in the literature, where most data on EIM/IMID in IBD are retrospective and specialist evaluations are inconsistently performed.^3^ TENOR illustrates the practical challenges of implementing the International Organization for the Study of Inflammatory Bowel Diseases EXTRA recommendations in routine clinical practice. UST’s therapeutic potential beyond intestinal disease warrants further prospective studies with optimized coordination and robust methodology.

## Supplementary Materials

Supplementary Material

Extended PDF
