# Supplementary material for: Ustekinumab for Extraintestinal Manifestations in Patients With Inflammatory Bowel Disease: Lessons From the TENOR Real-World Study
Source: Gastro Hep Adv. 2026 Sep 17;5(12):101115. doi: 10.1016/j.gastha.2026.101115 (PMC13681671; doi:10.1016/j.gastha.2026.101115)
Supplement: Supplementary file 1 [file mmc1.pdf]

Analysis set	Definition	Key criteria	Objective
**ESEAS** (Eligible Safety and Effectiveness Analysis Set)<br>N=109	Broad population including all eligible patients meeting main study inclusion criteria	- Fulfilled eligibility criteria<br>- No strict requirement for specialist-confirmed endpoint completion or timing constraints	- Describe overall patient population, treatment exposure, and safety outcomes in a broad population
**ESEAS-PP** (Eligible Safety and Effectiveness Analysis Set – Per protocol)<br>N=98	Same as ESEAS but with protocol visit windows applied (per-protocol timing).		
**EPEAS** (Effectiveness Per Eligibility Analysis Set)<br>N=76	Patients with specialist-confirmed EIM/IMID at baseline	- Specialist-confirmed EIM/IMID at baseline<br>- No strict constraints on timing of assessments<br>- Inclusion independent of complete follow-up timing windows	- Evaluate effectiveness in a real-world setting<br>- Maximize generalizability but may be subject to greater variability in endpoint assessment.
**EPEAS-PP** (Effectiveness Per Eligibility Analysis Set – Per protocol)<br>N=56	Same as EPEAS but with protocol visit windows applied (per-protocol timing).		
**PEAS** (Primary Effectiveness Analysis Set)<br>N=42	Subset of patients with more stringent clinical and data completeness requirements: moderate–severe IBD, baseline IBD assessment, baseline specialist confirmation, Week-24 specialist assessment, and SmPC-consistent ustekinumab exposure	- Moderate-to-severe IBD<br>- Available disease activity scores (HBI or PMS)<br>- Specialist-confirmed EIM/IMID<br>- More structured data collection	- Primary population for effectiveness analyses aligned with study objectives
**PEAS-PP** (Primary Effectiveness Analysis Set – Per protocol)<br>N=26	Highly selected subset of PEAS meeting strict methodological and temporal criteria (protocol visit windows applied)	- Validated time interval for IBD activity assessment (HBI/PMS)<br>- Defined interval between disease activity assessment and treatment initiation<br>- Validated and minimal interval for specialist-confirmed diagnosis at baseline<br>- Validated and minimal interval for specialist evaluation at Week 24	- Support sensitivity/robustness analyses of primary endpoint<br>- Ensure methodological consistency of endpoint assessment

Supplementary table 1: Details of the six complementary analysis populations.

Taken together, these complementary populations allow for a balanced interpretation of results, considering both internal validity and real-world generalizability. Compared with the EPEAS population, the PEAS-PP subset was defined using stricter methodological criteria, particularly regarding the temporal alignment between disease activity assessments and treatment initiation, as well as validated time windows for specialist evaluations at baseline and Week 24. Baseline demographic, clinical and treatment characteristics were overall comparable between the two populations, including age, sex distribution, type and duration of IBD, as well as a similar history of advanced disease with prior exposure to multiple biologics, notably anti-TNF agents, and frequent use of immunomodulators and corticosteroids. These additional constraints in PEAS-PP enhance the internal validity and reliability of outcome assessment. However, the limited sample size of the PEAS-PP subset precluded formal statistical analyses. Conversely, although EPEAS better reflects real-world practice, the lack of adherence to predefined inclusion criteria and assessment time windows limits the interpretability of the results and prevents drawing robust conclusions.
