# Supplementary material for: Ustekinumab for Extraintestinal Manifestations in Patients With Inflammatory Bowel Disease: Lessons From the TENOR Real-World Study
Source: Gastro Hep Adv. 2026 Sep 17;5(12):101115. doi: 10.1016/j.gastha.2026.101115 (PMC13681671; doi:10.1016/j.gastha.2026.101115)
Supplement: Supplementary file 2 [file mmc2.pdf]

Gastro Hep Advances 2026;5:101115

RESEARCH LETTER

Ustekinumab for Extraintestinal Manifestations in Patients With Inflammatory Bowel Disease: Lessons From the TENOR Real-World Study

Inflammatory bowel disease (IBD), encompassing Crohn's disease (CD) and ulcerative colitis (UC), is a chronic, immune-mediated inflammatory condition of the gastrointestinal tract.[1] Although gastrointestinal symptoms predominate, extraintestinal manifestations (EIMs) and immune-mediated inflammatory diseases (IMIDs) occur in 20% to 40% of patients, complicating management and reflecting a more complex, morbid disease phenotype.[2,3]

Biologic therapies have transformed IBD management, but evidence regarding their efficacy in EIM/IMID remains limited. Ustekinumab (UST), a monoclonal antibody targeting interleukin 12 and 23, is approved as second-line therapy for moderate-to-severe IBD. Emerging data suggest UST may confer benefits in EIM/IMID.[4–7] Assessment of EIM/IMID in clinical trials remains challenging, as emphasized by the EXTRA consensus from the International Organization for the Study of Inflammatory Bowel Diseases, which proposed standardized recommendations to harmonize evaluation and mitigate limitations.[8]

TENOR (EffecTiveness of ustEkinumab in patients with extra-iNtestinal manifestations associated with CrOhn's disease in a Real-world setting) was a prospective, multicenter, non-interventional French study conducted between October 2018 and November 2022 to assess UST impact on EIM/IMID in IBD through a multidisciplinary approach involving gastroenterologists and relevant specialists (dermatologists, rheumatologists, and ophthalmologists). Eligible adults had IBD, were initiating UST according to the French summary of product characteristics after prior anti-tumor necrosis factor therapy, and had at least one suspected active EIM/IMID.

Patients underwent evaluations at baseline (gastroenterologist and specialist), week 16 (gastroenterologist), and week 24 (gastroenterologist and specialist). EIM of interest included cutaneous (pyoderma gangrenosum, erythema nodosum), articular (axial and peripheral spondyloarthritis, dactylitis, enthesitis), and ocular (uveitis) manifestations. IMID comprised psoriasis, atopic dermatitis, and hidradenitis suppurativa.

The primary endpoint was the proportion of patients achieving clinical response (CR) for at least one EIM/IMID at week 24 as assessed by specialists. CR was evaluated using standardized tools. For dermatologic manifestations, CR was defined as a ≥2-point reduction in the Physician's Global Assessment score or a score of 0 to 1; erythema nodosum was assessed by lesion resolution. For articular involvement, CR was defined as a moderate or good response on Disease Activity Score in 28 joints for peripheral spondyloarthritis (≥0.6-point reduction), Bath Ankylosing Spondylitis Disease Activity Index50 or ≥2-point reduction for axial spondyloarthritis, and a 50% decrease or score of 0 on Leeds Enthesitis Index-6 for enthesitis. Dactylitis and uveitis were evaluated by lesion resolution. Secondary endpoints included UST efficacy on IBD, assessed using the Harvey–Bradshaw Index (HBI) for CD and the Partial Mayo Score (PMS) for UC.

The primary analysis used the Extended Primary Efficacy Analysis Set, comprising all enrolled patients with specialist-confirmed EIM/IMID and assessable primary endpoint data, irrespective of visit timing. Additional methodological details are available in the Supplementary Material. Descriptive analyses were performed using SAS version 9.4 (SAS Institute Inc, Cary, NC, USA).

A total of 109 patients were enrolled (mean age, 41.8 ± 13.7 years; female-to-male ratio, 2:1), of whom 89.9% had CD and 10.1% UC; 26.6% presented with severe disease. Mean disease duration was 12.9 ± 9.9 years for CD and 8.4 ± 5.3 years for UC. At baseline, mean HBI was 7.3 ± 3.7, and mean PMS was 4.0 ± 2.3. All patients had prior exposure to at least one anti-tumor necrosis factor. Concomitant therapy was reported in 25.7%, including 14.6% corticosteroids and 11.1% immunomodulators. The most frequent historical EIM/IMID were axial spondyloarthritis (40.4%), peripheral spondyloarthritis (33.0%), and psoriasis (16.5%). The mean time since the first EIM/IMID diagnosis was 6.6 ± 8.8 years (median, 2.5 years).

At baseline, 69.7% (76/109) of patients had specialist-confirmed EIM/IMID. Among these, the most common diagnoses were axial spondyloarthritis (47.4%; mean Bath Ankylosing Spondylitis Disease Activity Index, 6.5 ± 9.4), peripheral spondyloarthritis (27.6%; mean Disease Activity Score in 28 joints, 3.3 ± 0.9), psoriasis (22.4%; 35.3% moderate to severe), and enthesitis (9.2%; mean Leeds Enthesitis Index-6, 2.0 ± 1.4). Less frequent manifestations included atopic dermatitis (5.3%), hidradenitis suppurativa (4.0%), uveitis (4.0%), erythema nodosum (2.6%), dactylitis (2.6%), and pyoderma gangrenosum (1.3%).

At week 24, CR was evaluable in 52.3% (57/109) patients. Attrition was mainly due to lack of specialist confirmation at baseline (n = 33) and absence of week 24 specialist assessment (n = 18). Consequently, statistical analyses were restricted. Approaches for handling missing data across different analyses are detailed in Figure.

In Extended Primary Efficacy Analysis Set at week 24, 40.4% (23/76) achieved CR for at least one EIM/IMID. Response rates were higher for dermatologic (68.4%) than articular manifestations (21.1%). CR by manifestation is presented in Table.

Mean HBI and PMS scores decreased by 3.3 ± 4.0 and 2.6 ± 2.8

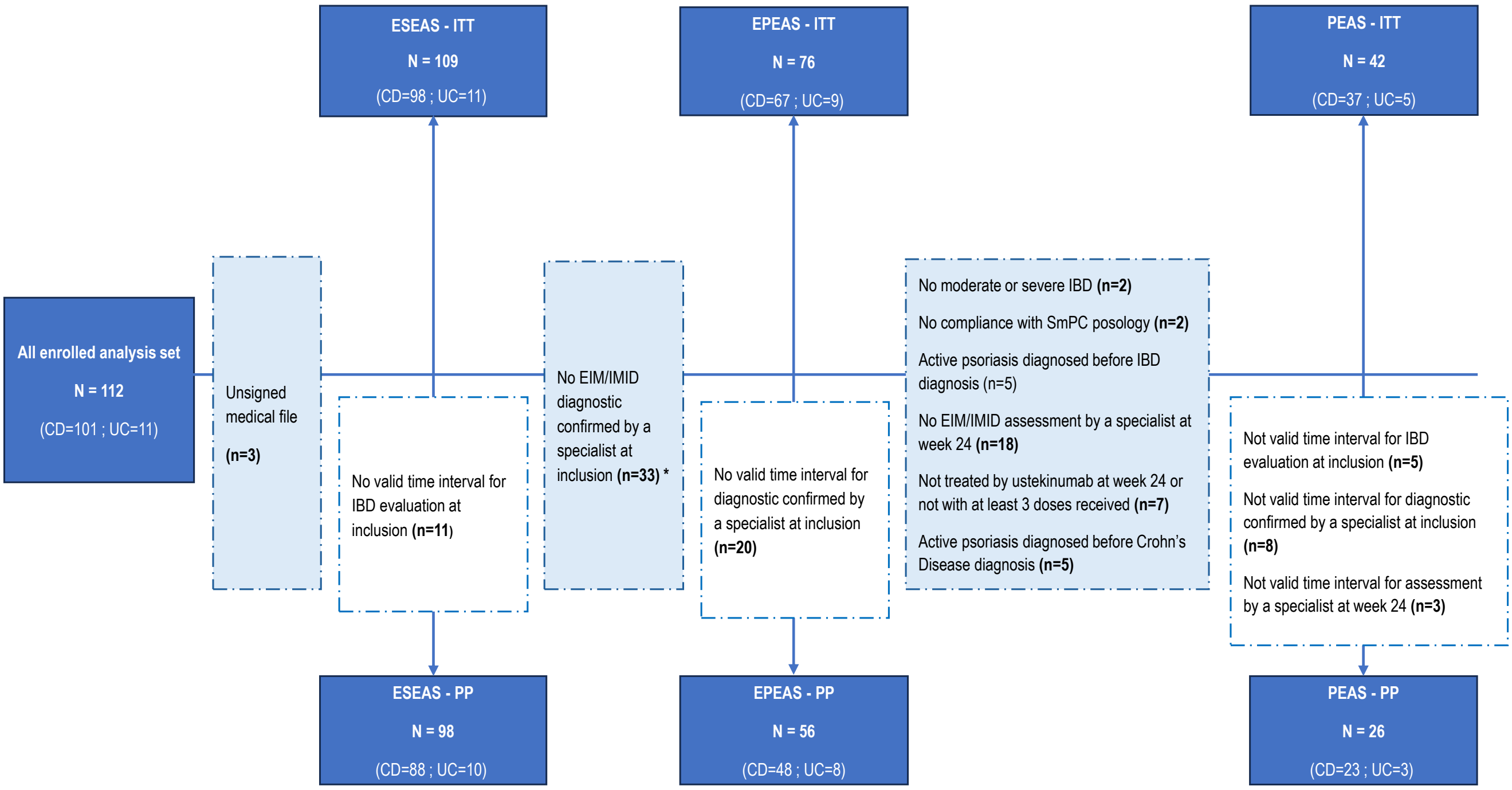

Figure. Definition of analysis sets (ITT and per protocol). EPEAS-ITT, Extended Primary Efficacy Analysis Set-intention-to-treat; EPEAS-PP, Extended Primary Efficacy Analysis Set-per protocol; ESEAS-ITT, Extended Secondary Efficacy Analysis Set-intention-to-treat; ESEAS-PP, Extended Secondary Efficacy Analysis Set-per protocol; PEAS-ITT, Primary Efficacy Analysis Set-intention-to-treat; PEAS-PP, Primary Efficacy Analysis Set-per protocol.

points, respectively. Clinical remission was achieved in 59.8% (58/97) of CD and 80.0% (8/10) of UC patients.

Overall, 49.5% (54/109) experienced at least one adverse event (AE). Treatment-related AEs occurred in 18 patients (16.5%) and serious AEs in 12.8%, including one treatment-related serious AE (arthralgia). Seven

Table. Effectiveness of Ustekinumab on the Different EIM/IMID Associated With IBD

		Week 24 assessment			
	Confirmed diagnosis at baseline, N	CR[a]<br>N N N	No CR	Missing data	UST effectiveness N (%)
Articular manifestations					
Axial spondyloarthritis	36	5	20	11	5/25 (20.0%)
Peripheral spondyloarthritis	22	3	13	6	3/16 (6.3%)
Enthesitis	8	2	1	5	2/3 (66.7%)
Dactylitis	2	0	1	1	0/1 (0.0%)
Cutaneous manifestations					
Psoriasis	17	9	4	4	9/13 (69.2%)
Atopic dermatitis	4	1	2	1	1/3 (33.3%)
Hidradenitis suppurativa	3	1	1	1	1/2 (50.0%)
Erythema nodosum	2	1	0	1	1/1 (100.0%)
Pyoderma gangrenosum	1	NA	NA	1	NA
Ophthalmic manifestations					
Uveitis	3	2	1	0	2/3 (66.7%)

EPEAS analysis.
DAS28, Disease Activity Score in 28 joints; EPEAS, Extended Primary Efficacy Analysis Set; LEI-6, Leeds Enthesitis Index; NA, not applicable; PGA, Physician's Global Assessment.
[a]CR defined for dermatological EIM PGA improved by 2 points or nil if initial PGA 1; erythema nodosum: resolution of lesions; peripheral spondyloarthritis: reduction >0.6 on DAS28; axial spondyloarthritis: Bath Ankylosing Spondylitis Disease Activity Index50 or ≥2 reduction in score; dactylitis: resolution of lesions; enthesitis: LEI-6 at 0 or 50% reduction in score; uveitis: resolution of lesions. Several EIM/IMID per patient possible.

patients (6.0%) discontinued UST due to an AE.

Although these findings suggest greater efficacy of UST in cutaneous IMID compared with articular manifestations as previously reported,[4] definitive conclusions cannot be drawn owing to limited data interpretability.

The TENOR study highlights the complexity of evaluating EIM/IMID in IBD. A major limitation of this study is the substantial amount of missing data, resulting in limited evaluability of the endpoints. Difficulties in obtaining timely specialist consultation for non-digestive symptoms contributed to missing assessments. Consequently, CR at week 24 could only be assessed in approximately half of the enrolled patients, which restricted the interpretability of findings. Strict assessment windows also contributed to substantial missing data in the per-protocol analysis.

The limited availability of specialist evaluations primarily reflects real-world challenges in coordinating multidisciplinary care, including limited access to specialists, constrained visit schedules, and the need for multiple assessments across heterogeneous EIM/IMID. Additional contributors included lack of specialist confirmation at baseline and missed follow-up evaluations.

In some cases, specialist evaluation did not corroborate the gastroenterologist's initial suspicion. The lack of standardized EIM/IMID definitions may limit clarity and comparability.

Study conduct was further challenged by the COVID-19 pandemic, which may have affected both patient access to care and completion of specialist assessments.

Consequently, analyses remained largely descriptive and should be interpreted with caution. Recruitment of rarer manifestations was limited, precluding meaningful interpretation for these conditions.

These difficulties mirror those reported in the literature, where most data on EIM/IMID in IBD are retrospective and specialist evaluations are inconsistently performed.[3] TENOR illustrates the practical challenges of implementing the International Organization for the Study of Inflammatory Bowel Diseases EXTRA recommendations in routine clinical practice. UST's therapeutic potential beyond intestinal disease warrants further prospective studies with optimized coordination and robust methodology.

ROMAIN ALTWEGG[1]
MICELI CORINNE[2]
PAUL CARLE[3]
SOSNOWIEZ KATIA[4]
BESSETTE CHRISTEL[5]
PEYRIN-BIROULET LAURENT[6]
[1]Department of Gastroenterology, Hôpital Saint-Eloi, CHU de Montpellier, Montpellier, France
[2]Department of Rheumatology, Cochin Hospital, Paris, France
[3]Department of Dermatology, CHU de Toulouse, Toulouse, France
[4]J&J Innovative Medicine, France
[5]ICON plc, Puteaux, France
[6]Department of Gastroenterology, CHRU de Nancy, Vandœuvre-lès-Nancy, France

Correspondence:
Address correspondence to: Romain Altwegg, MD, Department of Gastroenterology, Hôpital Saint-Eloi, CHU de Montpellier, 80 Avenue Augustin Fliche, 34090 Montpelliere, France. e-mail: r-altwegg@chu-montpellier.fr.

Supplementary Materials

Material associated with this article can be found, in the online version, at https://doi.org/10.1016/j.gastha.2026.101115.

References

1. Peyrin-Biroulet L, et al. Inflamm Bowel Dis 2011;17:471–478.
2. Vavricka SR, et al. Inflamm Bowel Dis 2015;21:1982–1992.
3. Guillo L, et al. United Eur Gastroenterol J 2020;8:1013–1030.
4. Guillo L, et al. J Crohns Colitis 2021;15:1236–1243.
5. Poddubnyy D, et al. Ann Rheum Dis 2014;73:817–823.
6. Blok JL, et al. Br J Dermatol 2016;174:839–846.
7. Khattri S, et al. Exp Dermatol 2017;26:28–35.
8. Guillo L, et al. Lancet Gastroenterol Hepatol 2022;7:254–261.

Abbreviations used in this paper: **AE, adverse event; CD, Crohn's disease; CR, clinical response; EIM, extraintestinal manifestation; HBI, Harvey–Bradshaw Index; IBD, inflammatory bowel disease; IMID, immune-mediated inflammatory disease; PMS, Partial Mayo Score; UC, ulcerative colitis; UST, ustekinumab**

Most current article

© 2026 The Author(s). Published by Elsevier Inc. on behalf of American Gastroenterological Association Institute. This is an open access article under the CC BY license (http://creativecommons.org/licenses/by/4.0/).
2772-5723
https://doi.org/10.1016/j.gastha.2026.101115

Received January 16, 2026. Accepted September 10, 2026.

Acknowledgments:
The authors thank all investigators for supporting the research: L. Alric, R. Altwegg, P. Andrau, Y. Badat, A. Benezech, Y. Bouhnik, A. Buisson, G. Cadiot, L. Caillo, L. Coimet Berger, P. Desreumaux, J. Filippi, M. Fumery, C. Gilletta, X. Hebuterne, F. Heluwaert, S. Kwiatek, D. Moussata, S. Nancey, L. Peyrin Biroulet, X. Roblin, I. Rosa, G. Saint Georges, JC. Saurin, P. Seksik, G. Sickersen, S. Viennot as well as the project team at Kappa Santé including N. Texier, M. Willaime, M. Coin Bavarot, T. Nedelec, and C. Leyravaud.

Conflicts of Interest:
The authors disclose the following: Romain Altwegg declares counseling, boards or fees for AbbVie, Alphasigma, Amgen, Biogen, Celltrion, Ferring, Janssen, Lilly, MSD, Pfizer, Sandoz, and Takeda. Miceli Corinne has received grants and served as a consultant for Novartis, Abbvie, Pfizer, MSD, and Roche and Janssen. Paul Carle has received grants and served as a consultant for AbbVie, Almirall, Amgen, Boehringer Ingelheim, Bristol Myers Squibb, Celgene, Eli Lilly, Janssen, Leo Pharma, Merck, Mylan, Novartis, Pfizer, Sandoz, and UCB. Sosnowiez Katia is an employee of J&J Innovative Medicine. Bessette Christel is an employee of ICON. Peyrin-Biroulet Laurent declares personal fees from Galapagos, AbbVie, Janssen, Genentech, Ferring, Tillots, Celltrion, Takeda, Pfizer, Index Pharmaceuticals, Sandoz, Celgene, Biogen, Samsung Bioepis, Inotrem, Allergan, MSD, Roche, Arena, Gilead, Amgen, BMS, Vifor, Norgine, Mylan, Lilly, Fresenius Kabi, OSE Immunotherapeutics, Enthera, Theravance, Pandion Therapeutics, Gossamer Bio, Viatris, and Thermo Fisher; grants from Abbvie, MSD, Takeda, Fresenius Kabi; and stock options from CTMA.

Funding:
J&J Innovative Medicine.

Ethical Statement:
The study protocol and amendments were reviewed by an independent Ethics Committee. The study was conducted in accordance with applicable regulatory requirements.

Data Transparency Statement:
Although these data are not currently publicly available for sharing, requests for sharing can be sent to the corresponding author and will be evaluated on an individual basis.

Reporting Guidelines:
STROBE (Strengthening the Reporting of Observational Studies in Epidemiology).

Gastro Hep Advances, Volume 5

Supplemental information

Ustekinumab for Extraintestinal Manifestations in Patients With Inflammatory Bowel Disease: Lessons From the TENOR Real-World Study

Romain Altwegg, Miceli Corinne, Paul Carle, Sosnowiez Katia, Bessette Christel, and Peyrin-Biroulet Laurent

Analysis set	Definition	Key criteria	Objective
**ESEAS** (Eligible Safety and Effectiveness Analysis Set)<br>N=109	Broad population including all eligible patients meeting main study inclusion criteria	- Fulfilled eligibility criteria<br>- No strict requirement for specialist-confirmed endpoint completion or timing constraints	- Describe overall patient population, treatment exposure, and safety outcomes in a broad population
**ESEAS-PP** (Eligible Safety and Effectiveness Analysis Set – Per protocol)<br>N=98	Same as ESEAS but with protocol visit windows applied (per-protocol timing).		
**EPEAS** (Effectiveness Per Eligibility Analysis Set)<br>N=76	Patients with specialist-confirmed EIM/IMID at baseline	- Specialist-confirmed EIM/IMID at baseline<br>- No strict constraints on timing of assessments<br>- Inclusion independent of complete follow-up timing windows	- Evaluate effectiveness in a real-world setting<br>- Maximize generalizability but may be subject to greater variability in endpoint assessment.
**EPEAS-PP** (Effectiveness Per Eligibility Analysis Set – Per protocol)<br>N=56	Same as EPEAS but with protocol visit windows applied (per-protocol timing).		
**PEAS** (Primary Effectiveness Analysis Set)<br>N=42	Subset of patients with more stringent clinical and data completeness requirements: moderate–severe IBD, baseline IBD assessment, baseline specialist confirmation, Week-24 specialist assessment, and SmPC-consistent ustekinumab exposure	- Moderate-to-severe IBD<br>- Available disease activity scores (HBI or PMS)<br>- Specialist-confirmed EIM/IMID<br>- More structured data collection	- Primary population for effectiveness analyses aligned with study objectives
**PEAS-PP** (Primary Effectiveness Analysis Set – Per protocol)<br>N=26	Highly selected subset of PEAS meeting strict methodological and temporal criteria (protocol visit windows applied)	- Validated time interval for IBD activity assessment (HBI/PMS)<br>- Defined interval between disease activity assessment and treatment initiation<br>- Validated and minimal interval for specialist-confirmed diagnosis at baseline<br>- Validated and minimal interval for specialist evaluation at Week 24	- Support sensitivity/robustness analyses of primary endpoint<br>- Ensure methodological consistency of endpoint assessment

Supplementary table 1: Details of the six complementary analysis populations.

Taken together, these complementary populations allow for a balanced interpretation of results, considering both internal validity and real-world generalizability. Compared with the EPEAS population, the PEAS-PP subset was defined using stricter methodological criteria, particularly regarding the temporal alignment between disease activity assessments and treatment initiation, as well as validated time windows for specialist evaluations at baseline and Week 24. Baseline demographic, clinical and treatment characteristics were overall comparable between the two populations, including age, sex distribution, type and duration of IBD, as well as a similar history of advanced disease with prior exposure to multiple biologics, notably anti-TNF agents, and frequent use of immunomodulators and corticosteroids. These additional constraints in PEAS-PP enhance the internal validity and reliability of outcome assessment. However, the limited sample size of the PEAS-PP subset precluded formal statistical analyses. Conversely, although EPEAS better reflects real-world practice, the lack of adherence to predefined inclusion criteria and assessment time windows limits the interpretability of the results and prevents drawing robust conclusions.
